# Hybridization between subterranean tuco-tucos (Rodentia, Ctenomyidae) with contrasting phylogenetic positions

**DOI:** 10.1038/s41598-020-58433-5

**Published:** 2020-01-30

**Authors:** Bruno Busnello Kubiak, Rafael Kretschmer, Leonardo Trindade Leipnitz, Renan Maestri, Thamara Santos de Almeida, Leandro Rodrigues Borges, Daniel Galiano, Jorge C. Pereira, Edivaldo Herculano Corrêa de Oliveira, Malcolm A. Ferguson-Smith, Thales Renato Ochotorena de Freitas

**Affiliations:** 1 Programa de Pós Graduação em Biologia Animal, Universidade Federal do Rio Grande do Sul, Av. Bento Gonçalves 9500, 91501-970 Porto Alegre, Brazil; 2 Programa de Pós-Graduação em Genética e Biologia Molecular, Universidade Federal do Rio Grande do Sul, Av. Bento Gonçalves 9500, 91501-970 Porto Alegre, Brazil; 3 Departamento de Ecologia, Universidade Federal do Rio Grande do Sul, Av. Bento Gonçalves 9500, 91501-970 Porto Alegre, Brazil; 4 Universidade Federal da Fronteira Sul, Campus Realeza, Rua Edmundo Gaievisk, 1000, CEP, 85770-000 Realeza, PR Brazil; 5 Cambridge Resource Centre for Comparative Genomics, Department of Veterinary Medicine, University of Cambridge, Cambridge, CB3 0ES UK; 6 Faculdade de Ciências Naturais, Instituto de Ciências Exatas e Naturais, Universidade Federal do Pará, Rua Augusto Correa, 01, Belém, Pará 66075-110 Brazil; 7 Laboratório de Cultura de Tecidos e Citogenética, Seção de Meio Ambiente, Instituto Evandro Chagas, BR-316, KM 7, s/n, 67030-000 Ananindeua, PA Brazil

**Keywords:** Phylogenetics, Cytogenetics, Evolutionary biology, Chromosomes, Animal physiology

## Abstract

Reproductive compatibility usually decreases according to increasing genetic difference and the time of divergence between species. However, the amount of modification required to influence hybridization may vary in different species. Thus, it is extremely important to conduct studies that seek to understand what and how variables influence the reproductive isolation of species. We have explored a system involving two species of subterranean rodents that present morphological, karyotypic, and evolutionary history differences and are capable of generating hybrids. To gain insight into the karyotype organization of genus *Ctenomys*, we examined the chromosome evolution by classical and molecular cytogenetics of both parental species and hybrids. Furthermore, we have used different approaches to analyze the differences between the parental species and the hybrids, and determined the origin of the hybrids. The results of our work demonstrate unequivocally that some species that present extensive differences in chromosome organization, phenotype, evolutionary history, sperm morphology and genetic, which are usually associated with reproductive isolation, can generate natural hybrids. The results also demonstrate that females of both species are able to generate hybrids with males of the other species. In addition, the chromosome-specific probes prepared from *Ctenomys flamarioni* provide an invaluable tool for comparative cytogenetics in closely related species.

## Introduction

The number of reports of hybrid animals has increased over the years^1^. Reproductive compatibility usually decreases according to increasing genetic difference and the time of divergence between species^2^. Some traits (e.g., genetic, karyotypic, morphological, and ecological) are related to the feasibility of hybridization between different species. However, the amount of modification required to influence hybridization may vary in different species influencing in the frequency of hybrid production and in the time of divergence that enables the generation of hybrids. For example, within vertebrates, mammals have the lowest hybridization rates^1,3^, and have evolved complete hybrid inviability on average faster than other vertebrates, such as birds^4,5^.

It is important to conduct studies that seek to understand what and how variables influence reproductive isolation. Normally, it is expected that mammals with distinct karyotypes are not capable of giving birth to hybrids; indeed, this is what is found in the majority of cases^6^. While this may be considered the normal pattern for mammals, it may also be the result of bias because of the difficulty in identifying hybrids^1^. In any case, hybrids have been documented in Eutherian mammals from parents with distinct chromosome numbers and karyotypes– for example, between the horse and the donkey, giving birth to the sterile mule^7^, which occurs artificially – and in Metatherian mammals at least two other cases have occurred^8^. In addition, there are cases in rodent races or subspecies in which hybrids are known between groups with different chromosome numbers^9–13^.

We have explored a system involving two species of subterranean rodents: *Ctenomys flamarioni* and *Ctenomys minutus*. The genus *Ctenomys* is the most specious among subterranean rodents, comprising approximately 70 described species^14^ with one of the highest rates of chromosomal variation among mammals from 2n = 10 to 2n = 70^15^. Parental species present several phenotypic differences: *C. minutus* has a predominantly brown, medium-dark hair color, with only the lower part of its body having a light brown coloration with shades of sand (see Supporting Information – Fig. S1). Populations of *C. minutus* have remarkable chromosomal variation, with six diploid numbers and eleven different karyotypes (2n = 42; 2n = 46a; 2n = 46b; 2n = 47a; 2n = 47b; 2n = 48a; 2n = 48b; 2n = 49a; 2n = 49b: 2n = 50a and 2n = 50b)^13,16,17^. *C*. *minutus* belongs to the *torquatus* species group^18^ and has single-tailed spermatozoa^19^. *C*. *minutus* also presents hybrid zones between chromosomally divergent populations^20^ and with *C*. *lami*, a phylogenetically closely related species^21^.

On the other hand, *Ctenomys flamarioni* has a predominantly white coloration (Fig. S1) and is morphologically more robust than the other *Ctenomys* species from southern Brazil^16,22^. The species belongs to the *mendocinus* species group^18^. In addition to their morphological similarity, they share the same chromosomal number (2n = 47–48), with the same chromosome G-band pattern among five species (*C. flamarioni, C. talarum, C. mendocinus, C. australis* and *C. porteousi*) and have an asymmetric, simple type of sperm with two tails^19,23–25^. *C*. *flamarioni* and *C. minutus* share one of the two sympatric zones described for the genus^26,27^, and in this region, we have information that they can generate hybrids. The common ancestor of *torquatus* and *mendocinus* was estimated to have arisen approximately 1.4 million years ago^28^.

The earlier comparative studies using conventional cytogenetic methods (Giemsa staining and chromosome banding pattern) have contributed to the establishment of chromosome homologies between some species of the genus *Ctenomys*^13,16,17^. However, cross-species chromosome painting (Zoo-FISH) allows a more accurate assessment of chromosomal rearrangements (e.g. translocations, tandem fusions and centric fusions or fissions) than traditional karyotype comparative techniques^29^. These chromosomal rearrangements are most likely to produce reproductive barriers when they cause problems at meiosis in heterozygotes, leading to reduced fertility^30^. The main problems at meiosis arise with translocations, tandem fusions and centric fusions or fissions, when a chromosome from one parental genome is homologous to two or more chromosomes from the other parental genome^30^. To gain insight into the karyotype organization of genus *Ctenomys*, we examined the chromosome evolution by classical and molecular cytogenetics of two species of this genus known to generate hybrids. In addition, we applied different approaches (cytogenetics, geometric morphometric and genetic analyses) to analyze the differences between the parental species and hybrids, and investigated the origin of the hybrids (i.e., by bidirectional breeding). Furthermore, we suggest that the chromosome-specific DNA probes for *C*. *flamarioni* generated here could become an invaluable tool for comparative cytogenetics in closely related species.

## Results

### Chromosome number and Ag-NOR patterns for parental and hybrid individuals

**Figure 1:**
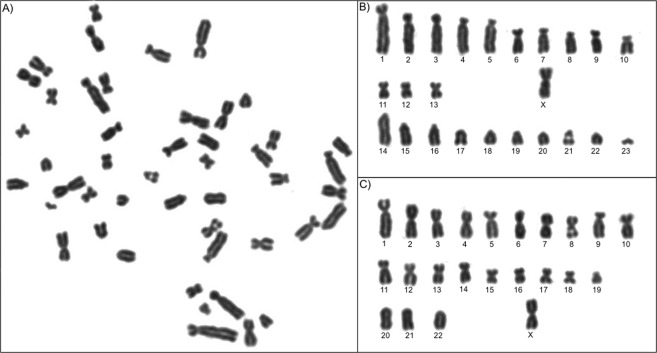
Karyotype characterization of a female hybrid individual between *Ctenomys minutus* (2n = 46) and *Ctenomys flamarioni* (2n = 48) using conventional Giemsa staining. Metaphase used in the characterization (**A**), haploid karyotypes from *Ctenomys flamarioni* (**B**) and *Ctenomys minutus* (**C**).

The chromosome number and structure for *C*. *minutus* (2n = 46) and *C*. *flamarioni* (2n = 48) confirm previous studies^16,24^. Hybrid individuals between these two species were identified for the first time and present a diploid number of 47, which is the sum of the haploid karyotypes of *C. minutus* (n = 23) and *C. flamarioni* (n = 24) (Fig. 1). Nucleolar organizer regions were found in two chromosomes, one metacentric and another acrocentric, in the respective haplotypes (see Supporting Information – Fig. S2).

### Flow karyotype of *C*. *flamarioni*

**Figure 2:**
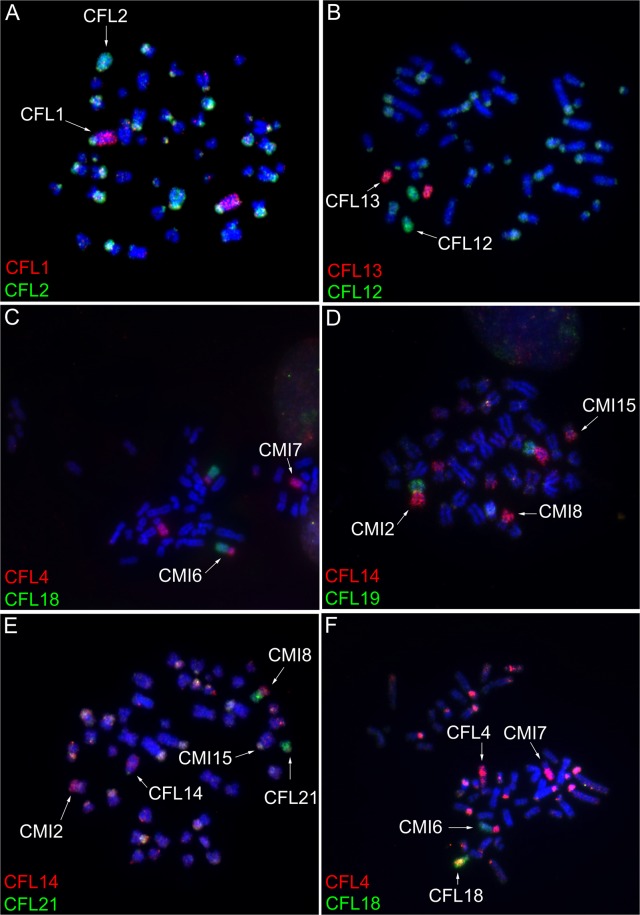
Representative FISH experiments using *Ctenomys flamarioni* (CFL) probes. Same-species hybridization (**A**,**B**) and cross-species chromosome painting on *Ctenomys minutus* metaphase chromosomes (CMI, **C**,**D**) and in a female hybrid individual (**E**,**F**). Biotin-CY3 (red) and digoxigenin-FITC (green).

The 48 chromosomes of one female *C. flamarioni* individual resolved into 22 peaks by flow cytometry (see Supporting Information - Fig. S3). The chromosomes in each peak of the flow karyotype were identified on *C. flamarioni* metaphases using FISH with labeled peak-specific DNA (Fig. 2). Pairs 20, 22 and 23 were contained in the same peak; however, the other chromosome pairs were separated individually. *C. flamarioni* had a high percentage of repetitive sequences, as previously described by de Freitas^24^ using C-banding. However, in all cases, it was possible to detect and identify chromosomes unequivocally.

### Comparative chromosome painting

**Figure 3:**
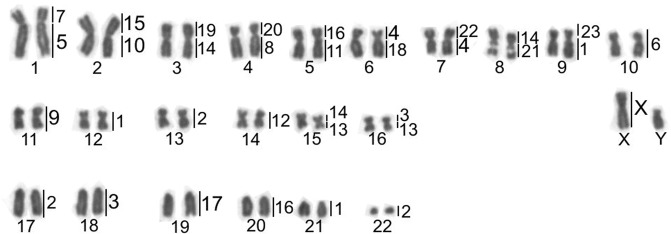
Karyotype of the *Ctenomys minutus* (2n = 46) showing homologies to *Ctenomys flamarioni* on the right of each chromosome pair of *Ctenomys minutus*.

The *C. flamarioni* chromosome-specific probes were hybridized to the metaphases of *C. minutus* and of the hybrid individuals between these two species. These hybridizations revealed that only four chromosomes of *C. flamarioni* (CFL6, 9, 12 and 17) have been fully conserved in *C. minutus* (CMI10, 11, 14 and 19). The other chromosomes are rearranged involving seven fissions and eleven fusions (Fig. 2). The regions of chromosomal homology between the two species are indicated in Fig. 3. Whole-chromosome painting probes of *C. flamarioni* in the hybrid individuals showed an entire haploid set of 23 chromosomes of *C. minutus* and 24 chromosomes of *C. flamarioni*.

### Mitochondrial DNA analysis: genetic distances

Genetic divergence between 33 Cyt *b* sequences was analyzed for a final data set containing 1,012 bp per sequence (out of 1,041 bp) after gaps and missing data were eliminated. Of the five hybrid individuals, four of them (TR1839, TR1854, TR1938 and TR1943) differed in over 5% of their sequences compared to the haplotypes assigned to the species *C. flamarioni* but differed between zero to 1.4% in sequence divergence compared to the haplotypes assigned to *C. minutus*; one hybrid individual (TR1844) differed between 4.3 to 5.4% of their sequences compared to the haplotypes assigned to the species *C. minutus*, but differed between zero and 0.1% compared to the haplotypes assigned to *C. flamarioni* (see Supporting Information - Table S1), irrespective of whether the haplotypes belonged to individuals sampled at Praia do Barco (Hybrid, BAR_fla and BAR_min) or elsewhere (GenBank vouchers CML 431, TR1215 and TR29). Hybrid individuals TR1839, TR1854, TR1938 and TR1943 presented identical Cyt b sequences, i.e., shared a haplotype – but differed in 5.2% of their sequences compared to TR1844.

Hybrid individuals TR1839, TR1854, TR1938 and TR1943 differed between 0.5 to 4% compared to the haplotypes of species within the *torquatus* species group (*C*. *torquatus*, *C*. *ibicuiensis*, *C*. *lami*, *C*. *pearsoni*, *C*. *perrensi*, *C*. *dorbignyi* and *C*. *roigi*) but differed between 4.4 to 5.3% compared to the haplotypes of species within the *mendocinus* species group (*C*. *australis*, *C*. *mendocinus*, *C*. *porteousi* and *C*. *rionegrensis*). All *Ctenomys* haplotypes differed in over 20% of their sequences compared to Octodontidae haplotypes (outgroups; *Spalacopus cyanus* and *Octodon degus*) (see Supporting Information - Table S2).

### Mitochondrial DNA analysis: molecular distance and phylogenetic analyses

**Figure 4:**
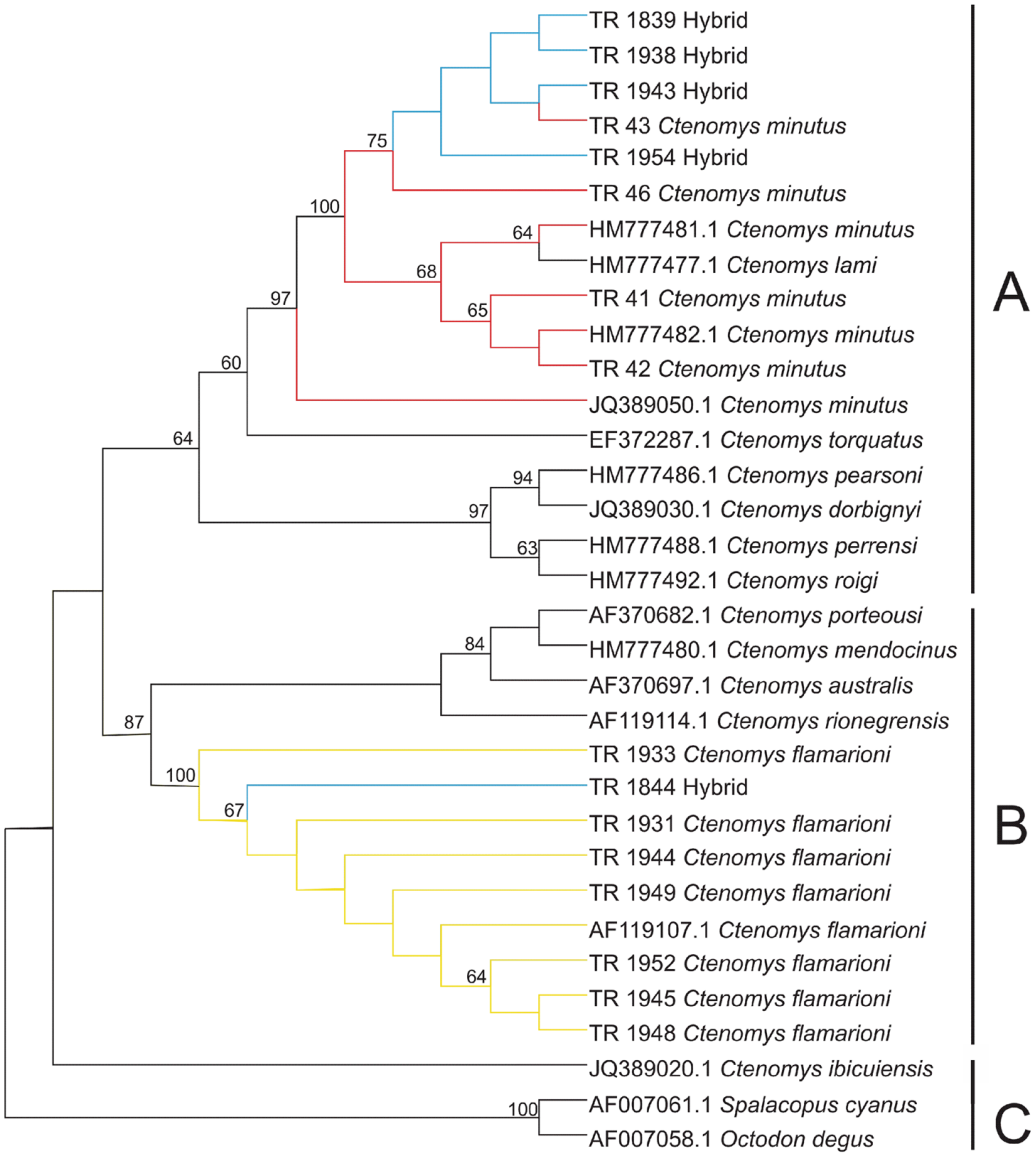
Phylogenetic analysis of 33 cytochrome b sequences (Cyt b; 1,041 bp) representative of the hybrid individuals, their parent species – *C. flamarioni* and *C. minutus* – and closely related species. The analysis was based on the Maximum Likelihood algorithm and the Hasegawa-Kishino-Yano (HKY) model of molecular evolution and considered gamma and invariant sites (G + I). (**A**) *Torquatus* species group; (**B**) *mendocinus* species group; (**C**) outgroup (Octodontidae).

The best-fit model of molecular evolution calculated by JModelTest2 was the Hasegawa-Kishino-Yano model with gamma and invariant sites (HKY + G + I; Hasegawa *et al*. 1985). Haplotypes used in the phylogenetic analysis cluster, as expected, were within one of two species groups – *torquatus* or *mendocinus* – with moderately strong to moderate statistical support (bootstrap values of 87 and 64, respectively). Hybrid individuals TR 839, TR1854, TR1938 and TR1943 clustered within the *torquatus* species group (group A, Fig. 4) alongside *C. torquatus*, *C. pearsoni*, *C. dorbignyi*, *C. perrensi*, *C. roigi* and other *C. minutus* haplotypes. Individual TR1844, on the other hand, clustered within the *mendocinus* species group together with haplotypes representative of the species *C. rionegrensis*, *C. porteousi*, *C. mendocinus*, *C. australis* and other *C. flamarioni* haplotypes.

### Microsatellite analysis

Estimates obtained through STRUCTURE and Structure Harvester analyses yielded different results. Structure Harvester estimated four genetic clusters (ΔK = 4) as the best K based on the Evanno method^31,32^.

**Figure 5:**
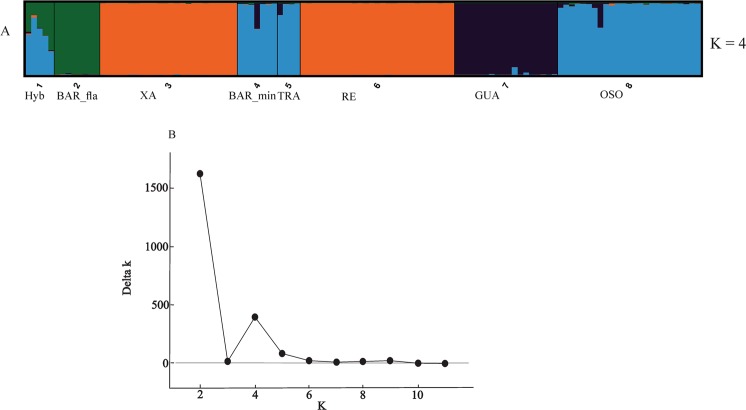
Bayesian based genetic clustering and specimen assignment for the clusters identified by Structure Harvester and STRUCTURE. Each specimen is represented by a single bar and each cluster by a color. Population Labels: Hyb - Hybrids; BAR_fla - *Ctenomys flamarioni* from Praia do Barco; XA - *Ctenomys flamarioni* from Xangri-lá; BAR_min – *Ctenomys minutus* from Praia do Barco; TRA – *Ctenomys minutus* from Tramandaí; RE - *Ctenomys minutus* from Remanso; GUA - *Ctenomys minutus* from Guarita; OSO - *Ctenomys minutus* from Osório. (**A**) Structure Harvester’s ΔK (K = 4). (**B**) Structure Harvester’s ΔK plot.

A K = 4 differentiates the eight populations into four genetic clusters. Populations of Xangri-la (XA) and Remanso (RE), both representative of parental *C. flamarioni*, cluster into a single, genetically homogeneous group (Fig. 5A, orange); accordingly, populations from Praia do Barco *C. minutus* (BAR_min), Tramandaí (TRA) and Osório (ORO), all representative of parental *C. minutus*, also cluster within a single genetic group (Fig. 5A, light blue). The Guarita (GUA) population, attributed to the species *C. minutus*, is isolated in a third homogeneous genetic cluster (Fig. 5A, purple). Lastly, the population of *C. flamarioni* from Praia do Barco (BAR_fla) clusters as a fourth genetically homogeneous group (Fig. 5A, green). The hybrid individuals (Hyb) present a mixed genetic composition with origins within the BAR_fla and BAR_min populations (Fig. 5A, light blue/green).

### Geometric morphometrics

**Figure 6:**
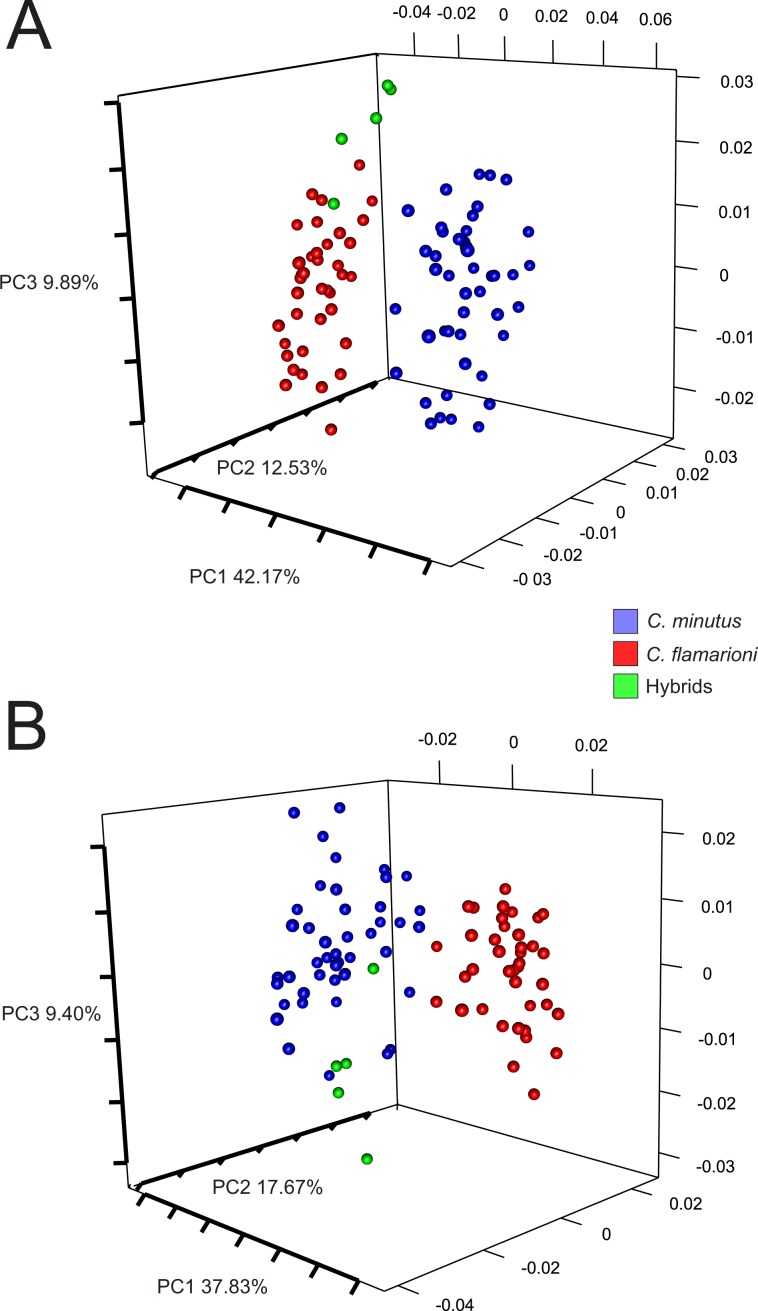
First three principal components of skull shape. (**A**) Dorsal and (**B**) ventral views of the skull.

Principal component analyses revealed a major axis of variation segregating individuals of the two parental species (Fig. 6). Hybrids aligned closest to *C. flamarioni* in dorsal shape but closest to *C. minutus* in ventral shape (Fig. 6A,B). In both cases, the third axis of variation showed a segregation of the hybrids from the parental species. This particular shape of hybrids was confirmed in discriminant analyses: percentages of correct classification based on a leave-one-out procedure indicated 100% correct classification for the three groups (*C. minutus*, *C. flamarioni*, and the hybrids) and for shape in both the dorsal and ventral views. The neighbor-joining trees again grouped all hybrid specimens closer together, but their relationship to each parental species was opposed depending on which skull view was considered: the hybrid group was closer to *C. flamarioni* in the dorsal view but closest to *C. minutus* in the ventral view (see supporting information - Figs. S4 and S5). The three approaches applied for shape converged in showing that hybrids had an intermediate shape relative to those of the parental species (closer to one or the other parental species depending on the skull view considered), suggesting a particular shape different with any parental species.

Centroid size variation showed that hybrids were bigger than *C. minutus* (see supporting information - Fig. S6). Statistically significant differences in size were recovered for dorsal (F = 22.12, *P* < 0.001) and ventral skull shape (F = 11.05, *P* < 0.001). However, the hybrids did not differ statistically in centroid size from *C. flamarioni* (dorsal P = 0.30, ventral P = 0.22). The similar size of hybrids to that of *C. flamarioni* was also evident after comparing the overall sizes of the specimens.

## Discussion

### Chromosomal organization in the hybrids and parental species

Subterranean rodents are characterized by extensive intra- and interspecific karyotypic variation, probably due to their life histories and habitat preferences^33^. For this reason, they have become important biological models in chromosome evolution^16,34,35^, generating the first mate preference experiments using mammals that hybridized naturally with *Spalax ehrenbergi*^10^; moreover, the early model for molecular characterization and analysis of hybrid zones was the pocket gopher^36–39^. One of the most fascinating cases is observed in *Ctenomys* species, which have 2n = 10 up to 2n = 70^15^. In addition, there are cases of hybridization between individuals of the same species with different cytotypes^13^. However, studies of chromosomal rearrangements in *Ctenomys* species and hybrid individuals to date have relied on traditional cytological techniques such as G-banding, which lack the resolution to detect small rearrangements. Hence, in this study we have developed an integrative approach to obtain insight into the hybrids between *C. minutus* (2n = 46) and *C. flamarioni* (2n = 48).

Previous studies showed that *C. minutus* (2n = 46) and *C. flamarioni* (2n = 48) presented differences in both chromosome morphology and diploid numbers^16,24^. Nucleolar organizer regions (NORs) are present in a medium metacentric chromosome in *C. minutus* (CMI8), whereas in *C. flamarioni* these regions are on a small acrocentric chromosome (CFL21)^16,24^. Thus, an F1 hybrid between these two species should have one NOR chromosome of *C. minutus* and one of *C. flamarioni*, which is exactly what was found. Therefore, our first evidence for the occurrence of hybridization between the two species was the NOR locations in addition to the diploid number of 47.

Chromosome-specific DNA probes developed from *C. flamarioni* allowed us to analyze the chromosomal complement of the hybrids and the parental species. All hybrid individuals had the same chromosomal organization and diploid number, indicating that they were F1 offspring. In addition, they were infertile as adult males did not produce mature spermatozoa. Despite the description of several hybrids among rodent species, in which chromosome painting has been performed on the parental species in a few cases, painting was not applied to the hybrids. There appear to be only two exceptions namely *Phodopus sungorus* and *P. campbelli*, which have the same chromosomal number (2n = 28)^40,41^, and *Microtus arvalis* (2n = 46) and *M. levis* (2n = 54), in which the difference in numbers is due to one fission and three fusions^29,42^. Thus, so far, hybrids of *C. minutus* and *C. flamarioni* demonstrate the greatest chromosomal reorganization among viable rodent hybrids. Species of the genus *Ctenomys* present high chromosomal variation (2n = 10 in *C*. *steinbachi* up to 2n = 70 for *C*. *pearsoni*)^15^, making this genus an excellent group for chromosome studies. Within the same species, variations also have been found; for example, *C*. *lami* presents 2n = 54 to 58^43^ and *C*. *minutus* with 2n = 42 to 50^16,17^. Therefore, the chromosome probes developed for *C*. *flamarioni* will certainly be valuable tools for understanding the evolution and origin of this chromosomal variability.

### Mitochondrial DNA and microsatellite variation in parental species and hybrid individuals

The mitochondrial DNA results demonstrate that hybridization occurs bidirectionally, i.e., that females of both species are able to generate hybrids with males of the other species, as the hybrids clustered with individuals of both *C*. *flamarioni* and *C*. *minutus* in a phylogenetic analysis. Indeed, genetic distances between the haplotypes of hybrid individuals and haplotypes representative of both species corroborated the phylogenetic analysis, as hybrids that cluster within *C*. *minutus* in the phylogeny are almost genetically indistinguishable from parental individuals of *C*. *minutus*, but present genetic distances greater than 4% when compared to parental individuals of *C*. *flamarioni*; the inverse reasoning can be applied to the hybrid individuals that cluster within *C*. *flamarioni*. This observation proves that hybridization between these species was not the result of error in species recognition by one of the females, which could be receptive to co-specific individuals. This phenomenon probably occurs due to the social organization of these individuals, where dominant males have access to several females^44,45^ with large home range areas^46^ and in this case do not distinguish females from the same or different species.

The microsatellite analysis results show that Evanno’s ΔK method (Fig. 5A, K = 4)considers the hybrid population to be consistently represented by genetic variation attributed to both parental populations (BAR_fla and BAR_min), indicating genetic admixture and a common genetic background for the hybrids. Additionally, these findings corroborate the geometric morphometrics analyses, in which morphological relatedness changed according to the observed view of the skull. The time of divergence of the most recent common ancestor for each species group has been estimated at 0.95 million years and 0.64 million years for the *torquatus* and *mendocinus* species groups, respectively, while common ancestors between both groups – which includes the *talarum* species group as well – were estimated to have originated approximately 1.4 million years ago^28^. This evidence indicates that the species *C*. *flamarioni* and *C*. *minutus* appeared within the expected age for mammals to have the ability to form hybrids^4,5^ and demonstrates that even genetically distant species with distinct evolutionary histories – and, possibly, different pre- and post-zygotic mechanisms of isolation – can generate hybrids within the genus *Ctenomys* if they come into contact.

### Morphological variation in parental species and hybrid individuals

Morphologically, hybrid individuals present a unique configuration. Geometric morphometric analysis shows that in the ventral view, the hybrids have characteristics similar to those of *C*. *minutus*, and in the dorsal view, they more closely resemble *C*. *flamarioni*. However, when viewed together, the hybrid individuals have characteristics that differ from those of both parental species, generating a unique morphological identity for the hybrids. Hybrid individuals were larger than parental individuals, although they did not differ statistically in skull size from *C*. *flamarioni*. This increase in size should be studied further, as it may be advantageous for individuals in possible interspecific interactions^47^. This is a pattern described for genera in which species inhabiting habitats with less hard soils (such as coastal dunes) have larger and more robust bodies compared to those of animals inhabiting harder soils^15^. The coat color of the animals also showed that they had characteristics of the parental species: although variable in color, the coat of these animals was darker than that of *C*. *flamarioni* and lighter than that of *C*. *minutus*. These differences should be studied in detail to test if such differences can bring adaptive advantages to the hybrids; for example, if differences in color can generate better camouflage and size competitive advantages.

## Conclusions and Prospects

The results of our work demonstrate unequivocally that, in some cases, species that present extensive chromosome organization, phenotype, evolutionary history, sperm morphology and genetics differences, which are usually associated with reproductive isolation, can generate natural hybrids. Furthermore, a series of findings in the field of ecology demonstrated that these two species present modifications during the occupation of microhabitats^27^ and morphological character displacement when in sympatry^48^, revealing that species are capable of recognizing individuals of another species and presenting ecological responses due to competition and yet they come into contact during the reproductive period and produce hybrids. Mitochondrial DNA analyses placed hybrid individuals within both species, thus providing evidence of bidirectional gene flow because females may belong to either species; furthermore, microsatellite analysis revealed that the genetic makeup of the hybrid population was the result of admixture between the two parental species.

Chromosome rearrangements still play a controversial role from the evolutionary point of view, particularly in *Ctenomys*^49^. Our results indicate that extensive chromosomal differences between parental species may not be sufficient to prevent reproduction between them; however, they may directly influence the fertility of the offspring (i.e., act as a postzygotic barrier). FISH results revealed that only four chromosomes of *C*. *flamarioni* (CFL6, 9, 12 and 17) have been fully conserved in *C*. *minutus* (CMI10, 11, 14 and 19), while the other chromosomes are rearranged by seven fissions and eleven fusions, which correspond to chromosome rearrangements usually realated to severe problems at meiosis in heterozygous^30^. However, specific analyses of chromosome synapsis and recombination in male and female hybrids are necessary to clarify the cytological basis of hybrid sterility between *C*. *flamarioni* and *C*. *minutus*. Furthermore, there are registered cases of subterranean rodents in which races and species with different karyotypes generate fertile hybrids^10,13,39,50,51^. This could mean that hybridization played an important role and future studies may take this information into account to better understand the evolutionary history of the group. That is, if species with as many differences as *C*. *flamarioni* and *C*. *minutus* can generate hybrids, it is possible that any two different species of *Ctenomys* may generate hybrids, regardless of genetic, karyotypic or ecological differences. However, there is currently no evidence that allows us to assume that these hybrids can be fertile. Furthermore, all hybrid males collected did not have spermatozoa. It is worth noting that the parental species have different types of spermatozoa: *C*. *flamarioni* presents spermatozoa with two tails (as do other members of the *mendocinus* group), and *C*. *minutus* presents spermatozoa with a simple tail (like other individuals of the genus)^19^. On the other hand, although females do not present any characteristics indicating that they are infertile. We cannot rule out the notion that the number of hybrids can be larger than the number shown here. This can easily be imagined if we predict that there is phenotypic variation in the studied hybrids and that these animals may bear resemblance to the parental individuals, to a greater or lesser extent. This concept can be extrapolated to the whole genus and to mammals in general, highlighting the need to pay more attention to animals with different characteristics. In addition, if hybrid individuals such as the ones described here occur in higher numbers than we imagine and possess some capacity to reproduce (either between hybrids themselves or with parental individuals), we should take serious care with *C*. *flamarioni* because of its conservation status (Endangered - ^52^). One of the main threats to the species is its restricted geographic distribution; therefore, if hybrid individuals occur in considerable numbers, they may become possible competitors to *C*. *flamarioni* and may occupy portions of the distribution range of the parental species. In this case, *C*. *flamarioni* may be more threatened than we imagine.

## Material and Methods

### Specimen capture

The first hybrid individual was accidentally caught during field activities in the region of sympatry between *C*. *flamarioni* and *C*. *minutus* (near Capão da Canoa/RS). After capture and verification that the individual differed in coloration relative to the parental species, the individual was subjected to chromosomal, morphological and genetic analyses to confirm its status as a hybrid. After confirming the specimen was a hybrid, other capture expeditions were undertaken. A total of five “pure” individuals of *C. flamarioni*, five “pure” individuals of *C. minutus* and five hybrid individuals were captured and used in the analyses performed in this study.

Specimens were captured using Oneida-Victor no. 0 Snap Traps and sacrificed in accordance with the guidelines of the American Society of Mammalogists’ Animal Care Committee^53^. All proceedings were approved by the Institutional Ethics Commission on Animal Use of the Universidade Federal do Rio Grande do Sul (project number: 28785), and all field procedures had the appropriate permissions from Brazil’s Environmental Agency (IBAMA, Authorization no. 14690-1).

### Metaphase preparations, diploid number and Ag-NOR

Chromosomes from a male individual of *C*. *minutus*, a female individual of *C*. *flamarioni* and from three hybrid individuals were obtained from fibroblast cultures, according to^54^, grown at 37 °C in Dulbecco’s Modified Eagle’s Medium - high glucose (Gibco) enriched with 15% fetal bovine serum (GIBCO), penicillin (100 units/ml) and streptomycin (100 mg/ml). Chromosome preparations were made following standard procedures, which included 1 hour in colchicine, 15 minutes in hypotonic solution (0.075 M KCl), and fixation in 3:1 methanol:glacial acetic acid. The diploid number and chromosome morphology for each individual were determined from at least 50 metaphase chromosomes stained with Giemsa 10% in 0.07 M phosphate buffer at pH 6.8, followed by air drying. Identification of chromosomes carrying the nucleolus organizer regions (NORs) was performed according to Howell and Black^55^.

### Flow sorting and generation of chromosome-specific probes

Chromosome preparations from a fibroblast cell line of a female *C*. *flamarioni* (CFL) were stained with Hoechst 33258 (2 μg/ml, Sigma) and chromomycin A3 (40 μg/ml, Sigma) and sorted on the basis of base pair composition and chromosome size. Chromosome suspensions of CFL were sorted on a dual-laser cell sorter (MoFlo, Beckman Coulter) at the Cambridge Resource Centre for Comparative Genomics, and approximately 400 chromosomes were sorted from each peak in the flow karyotype. Chromosome-specific paints for CFL were generated by DOP-PCR^56^. DOP-PCR-amplified chromosome-specific DNA was labeled with biotin-16-dUTP or digoxygenin-labeled dNTPs during secondary DOP-PCR amplification.

### Fluorescence *in situ* hybridization

Chromosome-specific painting of CFL in *C*. *minutus* (2n = 46) and in the hybrid individuals was performed as previously described^57^. The probes were denatured at 65 °C for 10 minutes and then preannealed by incubation at 37 °C for 30 minutes. Metaphase slides from fibroblast cultures were denatured by incubation in 70% formamide/2 X SSC solution at 65 °C for 1.3 minutes, quenched in ice-cold 70% ethanol, and dehydrated through a 70, 90, and 100% ethanol series. The preannealed paints were applied onto slides, covered with a coverslip, sealed with cow gum, and incubated for three days overnight at 37 °C. Posthybridization washes consisted of two 5-minutes incubations in 50% formamide at 40 °C followed by two 5-minutes incubations in 2 X SSC at the same temperature. Biotin-labeled probes were visualized using Cy3-avidin, while digoxygenin-labeled probes were visualized using FITC anti-rabbit. After detection, slides were mounted in Antifade containing DAPI.

### DNA extraction and mitochondrial DNA data: PCR amplification and sequencing

We analyzed 129 specimens representative of the species *C*. *flamarioni* Travi, 1989, *C*. *minutus* Nehring 1887 and five hybrid individuals for 8 microsatellite *loci*. All individuals were housed at Laboratório de Citogenética e Evolução, Departamento de Genética, Universidade Federal do Rio Grande do Sul, Porto Alegre, Brazil (see details in Table S3). The populations used in the microsatellite analysis were chosen to cover all known populations that may be closely related to the target hybrid population. Among these we have populations of both parental species in the same place where the hybrids were collected (Praia do Barco/RS) and in adjacent areas for *C. flamarioni* (Xangri-lá) and *C. minutus* (Guarita) (See Table S3). We also sequenced the cytochrome b gene (partial sequence, 1,041 bp) for captured individuals from a population of hybrids (Hybrid, n = 5), parental individuals of *C*. *flamarioni* (BAR_fla, n = 7), and parental individuals of *C*. *minutus* (BAR_min, n = 4), all from the Praia do Barco locality (Tables S3 and S4). Tissue samples were preserved in 70°GL alcohol and stored at -20 °C. DNA was extracted using the CTAB protocol^58^ with modifications. We checked sample quality by running 1.5% agarose gels and quantifying samples with a spectrophotometer (NanoDrop, ThermoFisher). Purified genomic DNA samples were diluted to a working concentration of 50 ng/uL and used in subsequent analyses.

The Cyt b gene was chosen for analyses because most information at the molecular level for *Ctenomys* is available for this gene only, which limits phylogenetic analysis between species from different species groups when using other *loci*. The PCR mix contained 100 ng (~2 µL) of purified genomic DNA, 0.4 µL of 10 mM forward and reverse primers, 0.4 µL of 10 mM deoxynucleotide triphosphates, 2.0 µL of 10X PCR buffer, 1.6 µL of 50 mM MgCl_2_ polymerase cofactor, and 0.2 µL of 5 U/uL DNA Taq polymerase (Ludwig Biotec), totaling 20 µL of reaction per sample. The PCR cycling conditions were as follows: initial denaturation at 94 °C for one minute, followed by 30 cycles of denaturation at 93 °C for one minute, primer annealing at 45 °C for one minute, and fragment extension at 72 °C for 1.5 minutes, ending with a final extension at 72 °C for 10 minutes. To confirm amplification, the PCR products were visualized in 1.5% agarose gel prior to sequencing. Sequencing was conducted abroad (Macrogen Inc., Seoul, Korea).

### Sequence divergence and phylogenetic analyses

A total of 33 sequences – 16 individuals sequenced de novo and 17 haplotypes representative of the *mendocinus* and *torquatus* species groups – were used in the sequence divergence and phylogenetic analyses. Of the 16 haplotypes downloaded from GenBank, one corresponds to an individual of *C*. *minutus* sampled at Praia do Barco (TR40, accession number: HM777482.1), two are representative of *C. minutus* of unknown origin (CML 431, accession number: HM777481.1; and TR1215, accession number: JQ389050.1), one corresponds to an individual of *C*. *flamarioni* of unknown origin (T29, accession number: AF119107.1), one corresponds to an individual of *C*. *ibicuiensis* (accession number: JQ389020.1), and 10 are representative of other species within the *mendocinus* and *torquatus* species groups (sensu 22); two other sequences of the family Octodontidae, which is a sister family to Ctenomyidae, were used as outgroups (Tables S3 and S4). We chose the aforementioned haplotypes because they are representative of all species within the parent species’ species groups and because those sequences were previously used in phylogenetic analyses in past studies and, therefore, are considered valid representatives of the species to which they are assigned in databases.

We aligned the sequences through the Muscle algorithm^59^ implemented in MEGA 6.0^60^ and estimated sequencing divergence between haplotypes using the Kimura two parameter (K2P) model, partitioning data into 1_st_, 2_nd_ and 3_rd_ codon positions and using 1,000 bootstrap replicates; all other parameters were held as default. We defined the evolutionary model to be used in the phylogenetic analysis using JModelTest 2^61,62^ and then proceeded to construct a phylogenetic tree based on the Maximum Likelihood algorithm using the software program MEGA 6.0, with data partitioned into 1_st_, 2_nd_ and 3_rd_ codon positions and using 1,000 bootstrap replicates, in order to place the hybrid individuals on a phylogenetic framework and to identify the maternal origin of each individual.

### Microsatellite data, PCR amplification and genotyping

We analyzed 118 individuals for 8 out of 14 microsatellite *loci* previously described in the literature (HAI primers^63^; SOC primers^64^). All *loci* are polymorphic and have been used in several studies to identify population structure and estimate population parameters of diversity, kinship and potential barriers to gene flow^13,65–68^. *Loci* for the populations Xangri-lá (XA), Remanso (RE), *C. minutus* Praia do Barco (BAR_min), Tramandaí (TRA), Osório (OSO) and Guarita (GUA) were scored in previous studies (*C. flamarioni*: XA and RE^69^; *C. minutus*: BAR_min, TRA, OSO and GUA^68^). We scored the same 8 *loci* to compare the population of *C. flamarioni* individuals from Praia do Barco (BAR_fla, n = 8) and the hybrid individuals (Hybrid, n = 5) through PCR amplification and subsequent genotyping. The PCR mix contained 100 ng (~2 µL) of purified genomic DNA, 0.4 µL of 10 mM forward and reverse primers, 0.2 µL of 10 mM deoxynucleotide triphosphates, 2.0 µL of 10X PCR buffer, 1.6 µL of 50 mM MgCl_2_ polymerase cofactor, and 0.2 µL of 5 U/µL DNA Taq polymerase (Ludwig Biotec), totaling 20 µL of reaction per sample. The PCR cycles were as follows: initial denaturation at 94 °C for five minutes, followed by 34 cycles of denaturation at 94 °C for 30 seconds, primer annealing at 55–62 °C for 30 seconds and fragment extension at 72 °C for 45 seconds, ending with a final extension at 72 °C for five minutes. The 5’ ends of each primer were marked with fluorescent dyes (Fam and HEX fluorescence) to allow for fragment genotyping. The PCR products were then visualized in 6% nondenaturing polyacrylamide gels to confirm amplification. Genotyping was conducted abroad (Macrogen Inc. Seoul, Korea).

### Microsatellite analysis

We tested all 118 individuals for population structure using STRUCTURE v2.3.4^70–72^, a software that performs Bayesian inference of population structure, suggesting the most likely number (the Natural Logarithm of the Probability of the data, or “Ln P(D)”, closest to zero) of genetic clusters (K) for a given data set, and estimates the genetic ancestry (Q) of each individual for a given number of genetic clusters (K). We tested our data with the following parameters: 1 to 15 genetic clusters (K = 1–15) computing five iterations for each K; for each iteration, 1,500,000 steps on a Markov Chain Monte Carlo were run, discarding 500,000 steps as burn-in. The ancestry and allele frequency models selected were the Admixture model and the Correlated Frequency model, respectively, since the individuals within the parental species and the hybrid individuals share a recent common ancestry. All other parameters were held as default.

Since increases in the significance of K may plateau for values lower than the best estimate of Ln P(D) and increase the variance of the data, the best “Ln P(D)” value does not necessarily correspond to the most biologically meaningful value; therefore, we tested the results obtained through STRUCTURE in Structure Harvester, a web browser application designed to visualize likelihood values of K obtained from STRUCTURE and estimate the value of K that causes the largest increase in information gain when simulating the number of clusters (that is, ΔK) while avoiding increases in the variance of the data (e.g., Evanno’s K^31,32^). We then plotted the best results for the estimation of Ks obtained through the analysis in STRUCTURE using CLUMPAK^73^.

### Correspondence between individuals and molecular markers

Individuals from the Hybrid and BAR_fla populations were directly compared for both nuclear and mitochondrial markers because all *loci* obtained for those individuals were sequenced and genotyped by the authors. Unfortunately, identification of the individuals from the BAR_min population genotyped for the microsatellite *loci* by their collection registry (TR numbers) was not possible because such individuals were identified by a different system in the microsatellite spreadsheet (kindly ceded by Lopes, CM^68^). However, it is important to point out that, even though they cannot be directly related, individuals from the BAR_min population sequenced for the cytochrome b gene in this study are among those analyzed through microsatellite markers in past studies^68^.

### Geometric morphometrics

We compared skull morphology among the 5 hybrids, 39 individuals of *C. flamarioni* and 45 specimens of *C. minutus*. Specimens of the parental species were taken from both allopatric and sympatric populations (*C. flamarioni-* 22 allopatric and 17 sympatric; *C. minutus-* 24 allopatric and 21 sympatric); these specimens were collected for a recent study^48^ and were deposited in the mammal collection of Laboratório de Citogenética e Evolução at Universidade Federal do Rio Grande do Sul. Since sexual dimorphism in skull shape and size is small for *C. minutus*^74^ and was assumed to be negligible, individuals from different sexes were pooled in all analyses. To investigate quantitative variations in size and shape among specimens, we collected two-dimensional images of the skulls of each specimen and then applied geometric morphometric techniques.

Skulls were photographed using a standard protocol^48^ and a Nikon P100 camera (3684 × 2736 resolution). Skull images were taken from the ventral and dorsal views. Based on Fornel *et al*.^74^, we selected 30 landmarks to digitize in the ventral view and 29 to digitize in the dorsal view^75^; digitization was performed using TPSDig 2 software^76^. Digitized landmarks composed a matrix that was subjected to a Generalized Procrustes Analysis to remove scale, positional, and orientation effects. The symmetric component was used to represent shape and the centroid size in mm^77^ to represent size. GPA was conducted in R^78^ using the package geomorph^79^.

We first explored variations in shape and size using PCA and boxplots, respectively, to discover apparent differences/resemblances among specimens. We classified the hybrids as a separate group from the parental species and used discriminant analysis of shape to reclassify individuals into groups using a leave-one-out procedure^80^. Differences in size among the three groups were investigated using an Analysis of Variance (ANOVA) for log centroid size to ascertain if hybrids are closely related to *C. minutus* or *C. flamarioni* or if they comprise a particular group with a skull form distinct from that of both parental species. Lastly, we calculated Procrustes distances among individuals and used this distance matrix to generate an unrooted neighbor-joining tree to visualize the morphological distances among hybrid specimens and the distances from hybrid specimens to all other individuals. Analyses and graphical visualizations were made in R (R Core Team 2018) with the packages geomorph^79^, rgl^81^, Morpho^82^, and ape^83^.

## Supplementary information

Supporting information.
