# Supplementary material for: Hybridization between subterranean tuco-tucos (Rodentia, Ctenomyidae) with contrasting phylogenetic positions
Source: Sci Rep. 2020 Jan 30;10:1502. doi: 10.1038/s41598-020-58433-5 (PMC6992752; doi:10.1038/s41598-020-58433-5)

**Hybridization between subterranean tuco-tucos (Rodentia, Ctenomyidae) with contrasting phylogenetic positions**

Bruno Busnello Kubiak, Rafael Kretschmer, Leonardo Trindade Leipnitz, Renan Maestri, Thamara Santos de Almeida, Leandro Rodrigues Borges, Daniel Galiano, Jorge C. Pereira, Edivaldo Herculano Corrêa de Oliveira, Malcolm A. Ferguson-Smith, Thales Renato Ochotorena de Freitas

**Supporting information**

Table S1: Pairwise genetic distances – in percentage points – between individual Cytochrome b (Cyt b; 1,041bp) sequences, based on the Kimura two parameter model (K2P). Included here are sequences representative of the species *Ctenomys minutus* and *Ctenomys flamarioni* only. Individuals by number: 1 – 5: *flamarioni* x *minutus* hybrids (Hybrid); 6 – 10: *C. minutus* from Praia do Barco (BAR_min); 11 – 12: *C. minutus* vouchers from GenBank (accession numbers: HM777481.1 and JQ389050.1); 13 – 20: *C. flamarioni* from Praia do Barco (BAR_fla); 21: *C. flamarioni* voucher from GenBank (accession number: AF119107.1).

|  | 1. | 2. | 3. | 4. | 5. | 6. | 7. | 8. | 9. | 10. | 11. | 12. | 13. | 14. | 15. | 16. | 17. | 18. | 19. | 20. |
| --- | --- | --- | --- | --- | --- | --- | --- | --- | --- | --- | --- | --- | --- | --- | --- | --- | --- | --- | --- | --- |
| 1.TR1839 | - |  |  |  |  |  |  |  |  |  |  |  |  |  |  |  |  |  |  |  |
| 2.TR1844 | 5.2 | - |  |  |  |  |  |  |  |  |  |  |  |  |  |  |  |  |  |  |
| 3.TR1854 | 0.0 | 5.2 | - |  |  |  |  |  |  |  |  |  |  |  |  |  |  |  |  |  |
| 4.TR1938 | 0.0 | 5.2 | 0.0 | - |  |  |  |  |  |  |  |  |  |  |  |  |  |  |  |  |
| 5.TR1943 | 0.0 | 5.2 | 0.0 | 0.0 | - |  |  |  |  |  |  |  |  |  |  |  |  |  |  |  |
| 6.TR40 | 0.2 | 5.2 | 0.2 | 0.2 | 0.2 | - |  |  |  |  |  |  |  |  |  |  |  |  |  |  |
| 7.TR41 | 0.2 | 5.2 | 0.2 | 0.2 | 0.2 | 0.0 | - |  |  |  |  |  |  |  |  |  |  |  |  |  |
| 8.TR42 | 0.2 | 5.2 | 0.2 | 0.2 | 0.2 | 0.0 | 0.0 | - |  |  |  |  |  |  |  |  |  |  |  |  |
| 9.TR43 | 0.0 | 5.2 | 0.0 | 0.0 | 0.0 | 0.2 | 0.2 | 0.2 | - |  |  |  |  |  |  |  |  |  |  |  |
| 10.TR46 | 0.2 | 5.4 | 0.2 | 0.2 | 0.2 | 0.4 | 0.4 | 0.4 | 0.2 | - |  |  |  |  |  |  |  |  |  |  |
| 11.CML431 | 0.4 | 5.2 | 0.4 | 0.4 | 0.4 | 0.2 | 0.2 | 0.2 | 0.4 | 0.6 | - |  |  |  |  |  |  |  |  |  |
| 12.TR1215 | 1.4 | 4.3 | 1.4 | 1.4 | 1.4 | 1.4 | 1.4 | 1.4 | 1.4 | 1.6 | 1.6 | - |  |  |  |  |  |  |  |  |
| 13.TR1931 | 5.2 | 0.0 | 5.2 | 5.2 | 5.2 | 5.2 | 5.2 | 5.1 | 5.2 | 5.4 | 5.2 | 4.3 | - |  |  |  |  |  |  |  |
| 14.TR1933 | 5.1 | 0.1 | 5.1 | 5.1 | 5.1 | 5.1 | 5.1 | 5.2 | 5.1 | 5.3 | 5.1 | 4.4 | 0.1 | - |  |  |  |  |  |  |
| 15.TR1944 | 5.2 | 0.0 | 5.2 | 5.2 | 5.2 | 5.2 | 5.2 | 5.3 | 5.2 | 5.4 | 5.2 | 4.3 | 0.0 | 0.1 | - |  |  |  |  |  |
| 16.TR1945 | 5.3 | 0.1 | 5.3 | 5.3 | 5.3 | 5.3 | 5.3 | 5.3 | 5.3 | 5.5 | 5.3 | 4.4 | 0.1 | 0.2 | 0.1 | - |  |  |  |  |
| 17.TR1948 | 5.3 | 0.1 | 5.3 | 5.3 | 5.3 | 5.3 | 5.3 | 5.3 | 5.3 | 5.5 | 5.3 | 4.4 | 0.1 | 0.2 | 0.1 | 0.0 | - |  |  |  |
| 18.TR1949 | 5.2 | 0.0 | 5.2 | 5.2 | 5.2 | 5.2 | 5.2 | 5.2 | 5.2 | 5.4 | 5.2 | 4.3 | 0.0 | 0.1 | 0.0 | 0.1 | 0.1 | - |  |  |
| 19.TR1952 | 5.3 | 0.1 | 5.3 | 5.3 | 5.3 | 5.3 | 5.3 | 5.3 | 5.3 | 5.5 | 5.3 | 4.4 | 0.1 | 0.2 | 0.1 | 0.0 | 0.0 | 0.1 | - |  |
| 20.TR29 | 5.3 | 0.1 | 5.3 | 5.3 | 5.3 | 5.3 | 5.3 | 5.3 | 5.3 | 5.5 | 5.3 | 4.4 | 0.1 | 0.2 | 0.1 | 0.2 | 0.2 | 0.1 | 0.2 | - |

Table S2: Genetic distances obtained for each hybrid individual and populations BAR_min and BAR_fla compared to haplotypes representative of species closely related to the species *Ctenomys flamarioni* and *Ctenomys minutus* (*torquatus* and *mendocinus* species groups, sensu Parada et al. 2011) based on the Kimura two parameter model (K2P). Values are shown in percentage points; only maximum values are shown when comparing populations with haplotypes. 1 – 5: hybrids; 6: *C. minutus* Praia do Barco; 7: *C. minutus* voucher from GenBank (HM777481.1); 8: *C. minutus* voucher from GenBank (JQ389050.1); 9: *C. flamarioni* Praia do Barco; 10: *C. flamarioni* voucher from GenBank (AF119107.1); 11 – 17: *torquatus* species group vouchers (accession numbers: Table S1); 18 – 21: *mendocinus* species group vouchers (accession numbers: Table S1); 22 – 23: Outgroups (family Octodontidae) (accession numbers: Table S1).

|  | 1. | 2. | 3. | 4. | 5. | 6. | 7. | 8. | 9. | 10. | 11. | 12. | 13. | 14. | 15. | 16. | 17. | 18. | 19. | 20. | 21. | 22. | 23. |
| --- | --- | --- | --- | --- | --- | --- | --- | --- | --- | --- | --- | --- | --- | --- | --- | --- | --- | --- | --- | --- | --- | --- | --- |
| 1.TR1839 | - |  |  |  |  |  |  |  |  |  |  |  |  |  |  |  |  |  |  |  |  |  |  |
| 2.TR1844 | 5.2 | - |  |  |  |  |  |  |  |  |  |  |  |  |  |  |  |  |  |  |  |  |  |
| 3.TR1854 | 0.0 | 5.2 | - |  |  |  |  |  |  |  |  |  |  |  |  |  |  |  |  |  |  |  |  |
| 4.TR1938 | 0.0 | 5.2 | 0.0 | - |  |  |  |  |  |  |  |  |  |  |  |  |  |  |  |  |  |  |  |
| 5.TR1943 | 0.0 | 5.2 | 0.0 | 0.0 | - |  |  |  |  |  |  |  |  |  |  |  |  |  |  |  |  |  |  |
| 6. BAR_min | 0.2 | 5.4 | 0.2 | 0.2 | 0.2 | - |  |  |  |  |  |  |  |  |  |  |  |  |  |  |  |  |  |
| 7. CML 431 | 0.4 | 5.2 | 0.4 | 0.4 | 0.4 | 0.2 | - |  |  |  |  |  |  |  |  |  |  |  |  |  |  |  |  |
| 8.TR1215 | 1.4 | 4.3 | 1.4 | 1.4 | 1.4 | 1.4 | 1.6 | - |  |  |  |  |  |  |  |  |  |  |  |  |  |  |  |
| 9.BAR_fla | 5.3 | 0.1 | 5.3 | 5.3 | 5.3 | 5.3 | 5.3 | 4.3 | - |  |  |  |  |  |  |  |  |  |  |  |  |  |  |
| 10. TR29 | 5.3 | 0.1 | 5.3 | 5.3 | 5.3 | 5.3 | 5.3 | 4.4 | 0.2 | - |  |  |  |  |  |  |  |  |  |  |  |  |  |
| 11. *C. torquatus* | 3.1 | 5.3 | 3.1 | 3.1 | 3.1 | 3.3 | 3.1 | 2.7 | 5.4 | 5.4 | - |  |  |  |  |  |  |  |  |  |  |  |  |
| 12. *C. ibicuiensis* | 4.1 | 5.9 | 4.1 | 4.1 | 4.1 | 4.3 | 4.5 | 3.8 | 6.1 | 6.1 | 4.1 | - |  |  |  |  |  |  |  |  |  |  |  |
| 13. *C. lami* | 0.5 | 5.5 | 0.5 | 0.5 | 0.5 | 0.7 | 0.3 | 1.7 | 5.5 | 5.6 | 3.2 | 4.6 | - |  |  |  |  |  |  |  |  |  |  |
| 14. *C. pearsoni* | 4.0 | 5.5 | 4.0 | 4.0 | 4.0 | 4.0 | 4.2 | 4.0 | 5.6 | 5.6 | 4.7 | 5.2 | 4.1 | - |  |  |  |  |  |  |  |  |  |
| 15. *C. perrensi* | 3.2 | 5.1 | 3.2 | 3.2 | 3.2 | 3.2 | 3.4 | 2.7 | 5.1 | 5.2 | 3.6 | 4.1 | 3.5 | 2.0 | - |  |  |  |  |  |  |  |  |
| 16. *C. dorbignyi* | 3.9 | 5.4 | 3.9 | 3.9 | 3.9 | 3.9 | 4.1 | 3.9 | 5.5 | 5.5 | 4.3 | 4.6 | 4.0 | 1.9 | 2.0 | - |  |  |  |  |  |  |  |
| 17. *C. roigi* | 3.9 | 5.5 | 3.9 | 3.9 | 3.9 | 4.1 | 4.1 | 3.5 | 5.6 | 5.6 | 4.2 | 4.7 | 4.2 | 3.5 | 1.9 | 3.4 | - |  |  |  |  |  |  |
| 18. *C. australis* | 4.6 | 2.4 | 4.6 | 4.6 | 4.6 | 4.9 | 4.3 | 4.6 | 2.5 | 2.5 | 4.7 | 5.3 | 5.0 | 5.0 | 5.0 | 4.7 | 5.0 | - |  |  |  |  |  |
| 19. *C. mendocinus* | 4.9 | 2.2 | 4.9 | 4.9 | 4.9 | 5.1 | 4.9 | 4.3 | 2.3 | 2.3 | 4.9 | 5.3 | 5.2 | 5.2 | 4.9 | 4.7 | 5.6 | 1.2 | - |  |  |  |  |
| 20. *C. porteousi* | 4.4 | 2.3 | 4.4 | 4.4 | 4.4 | 4.6 | 4.4 | 4.1 | 2.4 | 2.4 | 4.7 | 5.4 | 4.7 | 5.3 | 4.9 | 4.8 | 5.7 | 1.3 | 1.1 | - |  |  |  |
| 21. *C. rionegrensis* | 5.3 | 3.2 | 5.3 | 5.3 | 5.3 | 5.5 | 5.3 | 5.2 | 3.2 | 3.3 | 5.5 | 5.5 | 5.6 | 5.5 | 5.3 | 5.4 | 6.2 | 2.9 | 2.7 | 2.8 | - |  |  |
| 22. *S. cyanus* | 21.4 | 20.3 | 21.4 | 21.4 | 21.4 | 21.4 | 21.1 | 20.7 | 20.4 | 20.2 | 21.4 | 21.0 | 21.5 | 21.4 | 21.3 | 20.8 | 21.9 | 20.7 | 20.6 | 20.7 | 20.4 | - |  |
| 23. *O. degus* | 21.1 | 21.3 | 21.1 | 21.1 | 21.1 | 21.4 | 21.1 | 21.1 | 21.4 | 21.4 | 20.6 | 20.7 | 21.2 | 21.3 | 21.8 | 21.3 | 22.3 | 21.3 | 21.1 | 21.4 | 21.1 | 13.1 | - |

Table S3: General information on individuals used in the analyses. Left to right: Populations names and their abbreviations, species names, number of individuals used in the molecular analyses by marker – Cytochrome b (Cyt b) and microsatellite *loci* – and the reference study by marker. Accession numbers to the sequences produced in this study and to the sequences downloaded from data bases can be found in the supplemental material. fla = *Ctenomys flamarioni*, min = *Ctenomys minutus*.

| Population names (abbreviation) | Species names | No. of individuals |  |  | Reference study |  |
| --- | --- | --- | --- | --- | --- | --- |
|  |  | Cytochrome b (Cyt b) | Microsatellites (n = 8) |  | Cytochrome b | Microsatellites (n = 8) |
| *Ctenomys* hybrids (Hybrid) | *C. flamarioni* x *C. minutus* | N = 5 | N = 5 |  | This study | This study |
| Praia do Barco *flamarioni* (BAR_fla) | *C. flamarioni* | N = 7 | N = 8 |  | This study | This study |
| Praia do Barco *minutus* (BAR_min) | *C. minutus* | N = 4 | N = 7 |  | This study | Lopes and Freitas 2012 |
| Xangri-lá (XA) | *C. flamarioni* | N/A | N = 24 |  | N/A | Fernández-Stolz et al. 2007 |
| Remanso (RE) | *C. flamarioni* | N/A | N = 27 |  | N/A | Fernández-Stolz et al. 2007 |
| Tramandaí (TRA) | *C. minutus* | N/A | N = 4 |  | N/A | Lopes and Freitas 2012 |
| Guarita (GUA) | *C. minutus* | N/A | N = 18 |  | N/A | Lopes and Freitas 2012 |
| Osório (OSO) | *C. minutus* | N/A | N = 25 |  | N/A | Lopes and Freitas 2012 |
| Vouchers GenBank | Various species | N = 14 | N/A |  | Lessa and Cook, 1998; D’Elía et al. 1999; Parada et al. 2011 | N/A |
| Outgroups | *Spalacopus cyanus* and *Octodon degus* | N = 2 | N/A |  | Lessa and Cook, 1998 | N/A |
| TOTAL |  | N = 32 | N = 118 |  |  |  |

Table S4: Details about the individual sequences (Cytochrome b) used in the molecular analyses in this paper.

| Individual names | Species names | Species group (sensu Parada et al. 2011) | Accession numbers | Study references |
| --- | --- | --- | --- | --- |
| TR1839_Hybrid | *flamarioni* x *minutus* | - | MK452109 | This study |
| TR1844_Hybrid | *flamarioni* x *minutus* | - | MK452110 | This study |
| TR1854_Hybrid | *flamarioni* x *minutus* | - | MK452111 | This study |
| TR1938_Hybrid | *flamarioni* x *minutus* | - | MK452112 | This study |
| TR1943_Hybrid | *flamarioni* x *minutus* | - | MK452113 | This study |
| HM777482.1 C.minutus voucher TR 40 | *Ctenomys minutus* | *torquatus* | HM777482 | Parada et al. 2011 |
|  | *Ctenomys minutus* | *torquatus* |  | This study |
| TR41_C.minutus | *Ctenomys minutus* | *torquatus* | MK452114 | This study |
| TR42_C.minutus | *Ctenomys minutus* | *torquatus* | MK452115 | This study |
| TR43_C.minutus | *Ctenomys minutus* | *torquatus* | MK452116 | This study |
| TR46_C.minutus | *Ctenomys minutus* | *torquatus* | MK452117 | This study |
| HM777481.1 C.minutus voucher CML 431 | *Ctenomys minutus* | *torquatus* | HM777481 | Parada et al. 2011 |
| JQ389050.1 C.minutus voucher TR1215 | *Ctenomys minutus* | *torquatus* | JQ389050 | Freitas et al. 2012 |
| HM777477.1_C.lami | *Ctenomys lami* | *torquatus* | HM777477 | Parada et al. 2011 |
| HM777486.1_C.pearsoni | *Ctenomys. pearsoni* | *torquatus* | HM777486 | Parada et al. 2011 |
| HM777488.1_C.perrensi | *Ctenomys perrensi* | *torquatus* | HM777488 | Parada et al. 2011 |
| EF372287.1_C.torquatus | *Ctenomys torquatus* | *torquatus* | EF372287 | Gonçalves and Freitas, 2009 |
| JQ389030.1_C.dorbignyi | *Ctenomys dorbignyi* | *torquatus* | JQ389030 | Freitas et al. 2012 |
| HM777492.1_C.roigi | *Ctenomys roigi* | *torquatus* | HM777492 | Parada et al. 2011 |
| TR1931_C.flamarioni | *Ctenomys flamarioni* | *mendocinus* | MK452118 | This study |
| TR1933_C.flamarioni | *Ctenomys flamarioni* | *mendocinus* | MK452119 | This study |
| TR1944_C.flamarioni | *Ctenomys flamarioni* | *mendocinus* | MK452120 | This study |
| TR1945_C.flamarioni | *Ctenomys flamarioni* | *mendocinus* | MK452121 | This study |
| TR1948_C.flamarioni | *Ctenomys flamarioni* | *mendocinus* | MK452122 | This study |
| TR1949_C.flamarioni | *Ctenomys flamarioni* | *mendocinus* | MK452123 | This study |
| TR1952_C.flamarioni | *Ctenomys flamarioni* | *mendocinus* | MK452124 | This study |
| AF119107.1 C.flamarioni T 29 | *Ctenomys flamarioni* | *mendocinus* | AF119107 | D’Élia et al. 1999 |
| AF370697.1 C.australis | *Ctenomys australis* | *mendocinus* | AF370697 | Slamovits et al. 2001 |
| HM777480.1_C.mendocinus | *Ctenomys mendocinus* | *mendocinus* | HM777480 | Parada et al. 2011 |
| AF370682.1 C.porteousi | *Ctenomys porteousi* | *mendocinus* | AF370682 | Slamovits et al. 2001 |
| AF119114.1_C.rionegrensis | *Ctenomys rionegrensis* | *mendocinus* | AF119114 | D’Élia et al. 1999 |
| AF007061.1_S.cyanus | *Spalacopus cyanus* | Outgroup | AF007061 | Lessa and Cook, 1998 |
| AF007058.1_O.degus | *Octodon degus* | Outgroup | AF007058 | Lessa and Cook, 1998 |

Figure S1. Images of parental species and hybrids. A - *Ctenomys minutus*, B - hybrid and C - *Ctenomys flamarioni.*

**
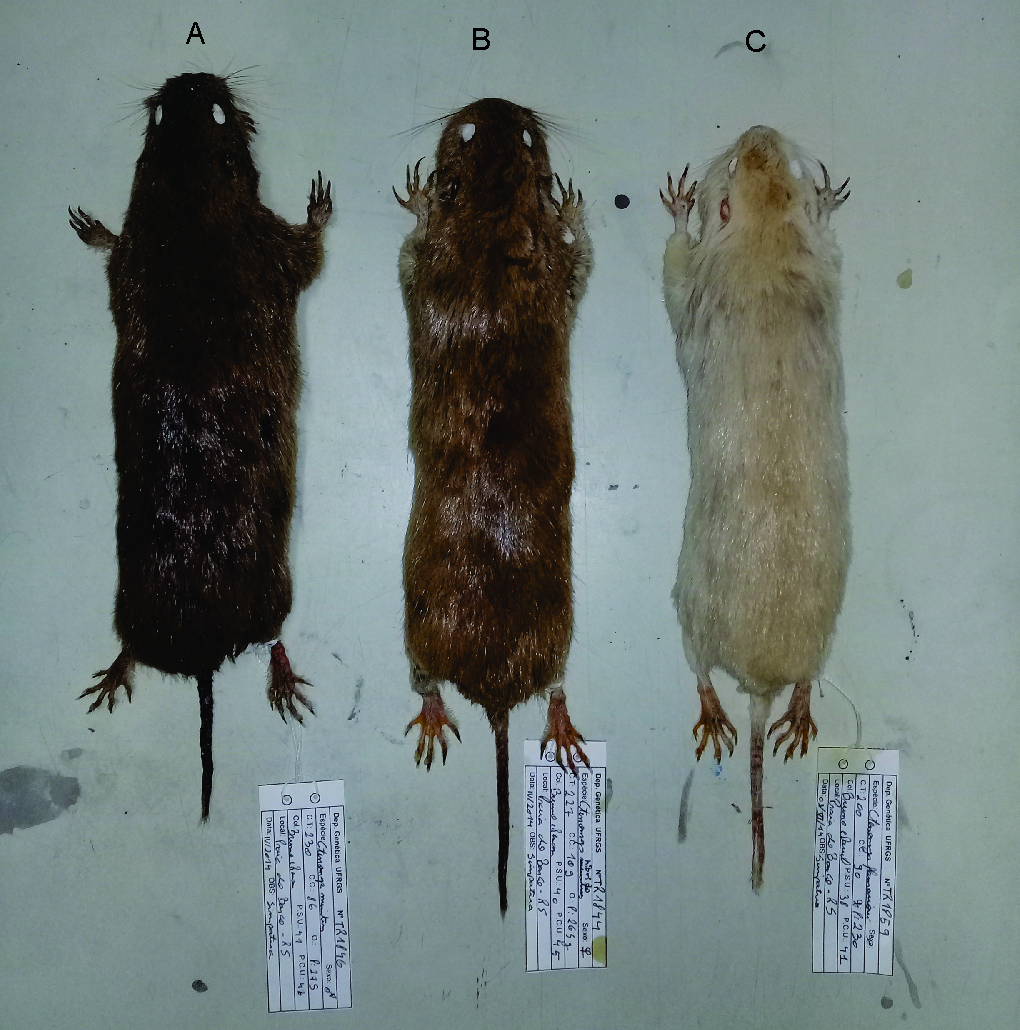
**

Figure S2 - Complete haploid karyotype of a female hybrid individual between *Ctenomys flamarioni* (A) and *Ctenomys minutus* (B) in AgNOR staining.


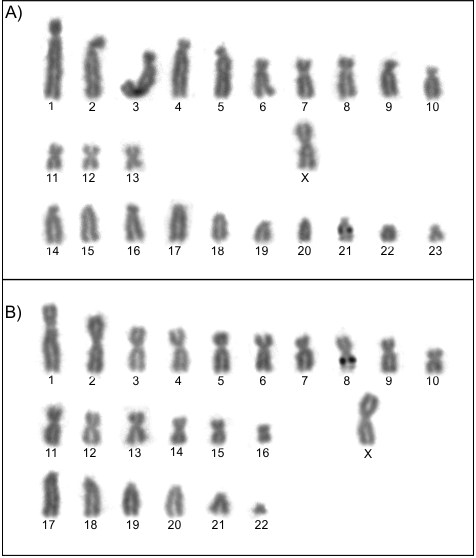


Figure S3. Bivariate flow karyotype of *Ctenomys flamarioni* (2n = 48) with chromosome assignments.


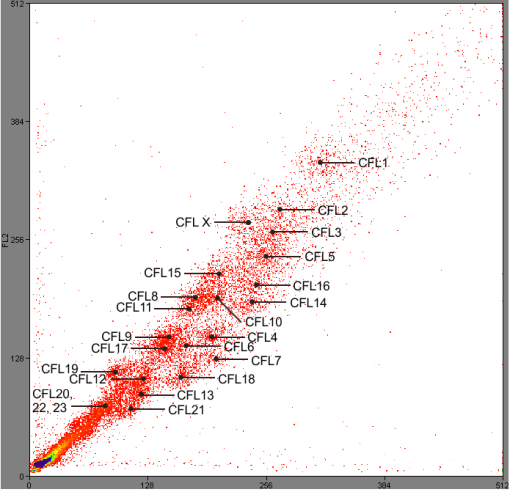


Figure S4 - Neighbor joining tree based on the Procrustes distance of ventral view from individuals of *Ctenomys flamarioni* (red squares), *Ctenomys minutus* (blue squares) and hybrids’ (green squares) skulls.


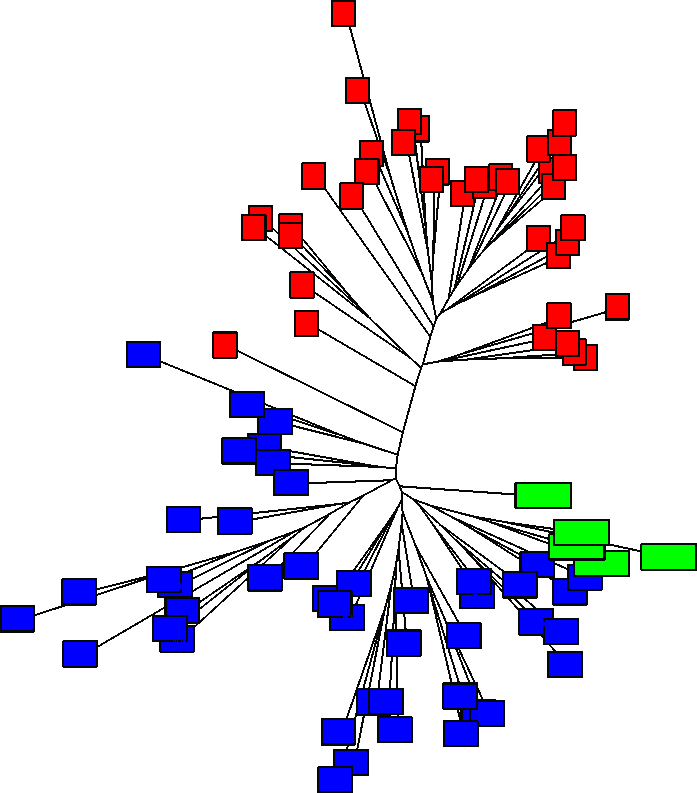


Figure S5 - Neighbor joining tree based on the Procrustes distance of dorsal view from individuals of *Ctenomys flamarioni* (red squares), *Ctenomys minutus* (blue squares) and hybrids’ (green squares) skulls.


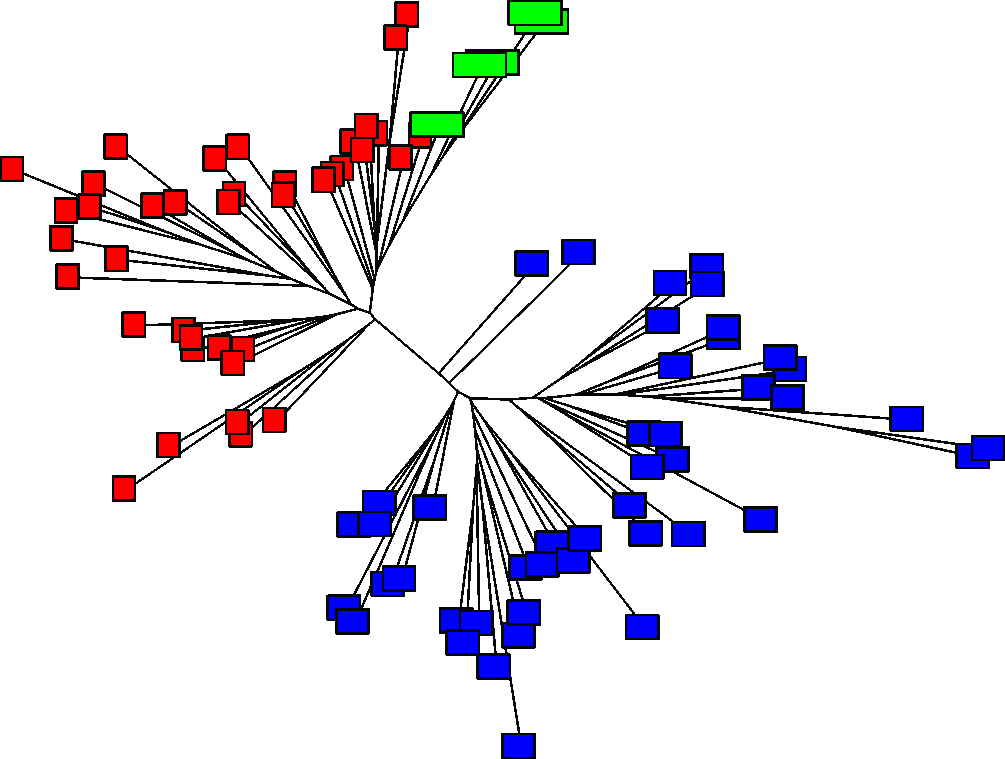


Figure S6. Boxplots showing variation in skull centroid size calculated from A. dorsal and B. ventral views of the skull.


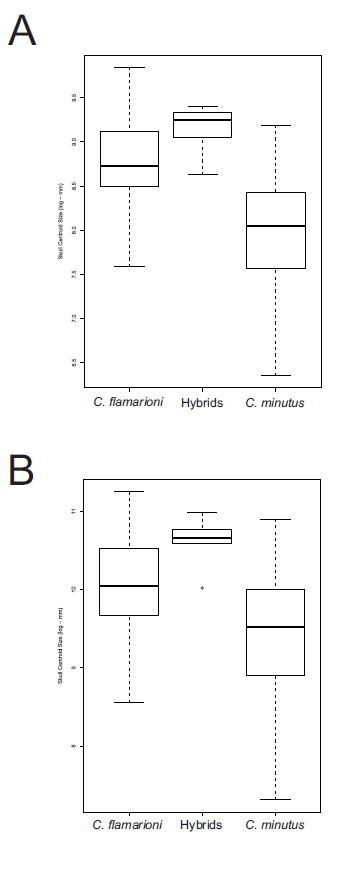

Supplement: Supplementary file 1 — Supporting information. [file 41598_2020_58433_MOESM1_ESM.docx]
